# Beyond Carrier Design: Fabrication Method as the Hidden Driver of NSAID Nanomedicine Performance

**DOI:** 10.3390/pharmaceutics18070877

**Published:** 2026-07-17

**Authors:** Ana-Maria Raluca Pauna, Liliana Mititelu-Tartau, Angy Abu Koush, Roxana Ionela Vasluianu, Jamal Al Ashkar, Ruxandra Teodora Stan, Viorel Radu, Marius Constantin Moraru, Cosmin Gabriel Popa, Roxana Florentina Gavril, Dragos Valentin Crauciuc, Andreea Ludusanu, Cristinel Ionel Stan, Alin Mihai Vasilescu

**Affiliations:** “Grigore T. Popa” University of Medicine and Pharmacy, Universitatii No. 16 Street, 700115 Iasi, Romania; ana-maria.pauna@umfiasi.ro (A.-M.R.P.); maierean_angy@yahoo.com (A.A.K.); roxana.vasluianu@umfiasi.ro (R.I.V.); jamal.al-ashkar@d.umfiasi.ro (J.A.A.); ruxandra-teodora.stan@umfiasi.ro (R.T.S.); viorel.radu@yahoo.com (V.R.); mariusmmc@gmail.com (M.C.M.); cosmin-gabriel.popa@umfiasi.ro (C.G.P.); roxi.sufaru@yahoo.com (R.F.G.); crauciuc.dragos@gmail.com (D.V.C.); andreea.ludusanu@umfiasi.ro (A.L.); cristinel.stan@umfiasi.ro (C.I.S.); alin.vasilescu@umfiasi.ro (A.M.V.)

**Keywords:** diclofenac, NSAIDs, nanomedicine, drug delivery systems, nanocarriers, microfluidics, nanostructured lipid carriers, Quality by Design, encapsulation efficiency, reproducibility, scale-up

## Abstract

**Background/Objectives:** Diclofenac (DCF) and other nonsteroidal anti-inflammatory drugs (NSAIDs) are widely used for pain and inflammation management; however, their clinical significance is limited by poor aqueous solubility, short biological half-life, and dose-dependent gastrointestinal, renal, and cardiovascular adverse effects. Nanocarrier-based delivery systems have been extensively explored because they can enhance the apparent solubility of poorly water-soluble NSAIDs, provide controlled and sustained drug release, prolong systemic circulation, and improve drug localization at the site of action. By reducing peak plasma concentrations and off-target exposure, these systems may decrease dose-dependent gastrointestinal and systemic adverse effects while maintaining therapeutic efficacy. Most studies focus on optimizing formulation composition, while the manufacturing process is often treated as a secondary parameter. The research critically evaluates conventional and emerging fabrication methods for NSAID nanocarriers, using DCF as the principal reference compound, with emphasis on their impact on physicochemical characteristics, reproducibility, scalability, and translational potential. **Methods:** A structured literature search was performed in PubMed/MEDLINE, Scopus, and Web of Science (2015–2026, with emphasis on 2022–2026) for DCF and NSAID-loaded submicron delivery systems reporting quantitative formulation data and clearly defined fabrication methods, resulting in a narrative review of approximately 375–395 eligible studies, comprising 75 DCF-specific studies and approximately 300–320 studies involving other NSAIDs that were included as representative surrogate systems when DCF-specific evidence was unavailable for particular fabrication approaches. The review followed Scale for the Assessment of Narrative Review Articles (SANRA) recommendations. Studies were analyzed using a standardized seven-parameter framework including encapsulation efficiency, release profile, particle size control, polydispersity, scalability, reproducibility, and process complexity. **Results:** Batch-based techniques, such as thin-film hydration for chitosan-coated liposomal systems, consistently provide high encapsulation efficiency, sustained drug release, and good biocompatibility. However, these methods are often associated with batch-to-batch variability, operator dependence, and limited scalability. In contrast, continuous manufacturing approaches, including microfluidic mixing, nanostructured lipid carriers, and Quality-by-Design (QbD)–guided processes, demonstrate improved control over particle size distribution and polydispersity, enhanced reproducibility, and better scalability potential. **Conclusions:** Manufacturing methodology is an important determinant of DCF and NSAID nanocarrier performance alongside formulation composition. Continuous manufacturing approaches offer promising improvements in reproducibility, process control, and scalability, but current evidence remains uneven across different nanocarrier classes. Further standardized comparative studies are needed to support their broader translation into clinical applications.

## 1. Introduction

NSAIDs represent one of the most widely used pharmacological classes in clinical practice. Among them, DCF has become one of the most extensively investigated model compounds for nanocarrier-based drug delivery systems owing to its widespread clinical use in the management of pain and inflammation associated with rheumatoid arthritis, osteoarthritis, and acute musculoskeletal conditions, as well as its well-recognized biopharmaceutical and safety limitations [1,2,3,4]. Despite its established therapeutic efficacy, DCF use is limited by well-documented safety concerns. As a non-selective cyclooxygenase inhibitor, it is associated with dose- and duration-dependent gastrointestinal toxicity, as well as increased cardiovascular and renal risks upon prolonged administration [1,5,6]. These limitations, together with its poor aqueous solubility, short plasma half-life, and requirement for repeated dosing, have driven continuous efforts toward the development of advanced drug delivery systems capable of improving its therapeutic index [3,7,8].

Although DCF is used as the principal reference compound throughout this review, similar biopharmaceutical and safety challenges are shared by several widely prescribed NSAIDs. Many members of this therapeutic class exhibit poor aqueous solubility, limited oral bioavailability, frequent dosing requirements, and dose-dependent gastrointestinal, renal, and cardiovascular adverse effects, thereby providing a common rationale for the development of nanocarrier-based delivery systems. Representative examples are summarized in Table 1.

Among the explored strategies, nanoencapsulation has emerged as a particularly effective approach to enhance the biopharmaceutical performance of DCF and related NSAIDs. Encapsulation within nanoscale carriers can improve the apparent solubility of poorly water-soluble NSAIDs, protect the drug from premature degradation, enable controlled and sustained drug release, and prolong systemic circulation. These properties help maintain therapeutic drug concentrations over longer periods while reducing fluctuations in plasma levels. In addition, by improving drug localization at the target tissue and reducing nonspecific exposure of healthy organs, nanocarriers may decrease dose-dependent gastrointestinal, renal, and cardiovascular adverse effects commonly associated with conventional NSAID therapy [9,10]. A broad range of nanocarrier systems has been reported in the literature, including polymer-based nanoparticles such as poly(lactic-co-glycolic acid) (PLGA) systems, as well as lipid-based vesicular and hybrid lipid–polymer platforms [11,12,13,14]. In particular, chitosan-coated lipid systems have been investigated for their ability to combine sustained release properties with enhanced biocompatibility and reduced gastrointestinal irritation compared with the free drug [15,16]. Across these formulations, parameters such as particle size, polydispersity index (PDI), zeta potential, and encapsulation efficiency are consistently considered critical quality attributes defining nanocarrier performance [9].

However, despite the large body of research focusing on material selection and carrier optimization, the fabrication process itself is often underexplored and treated as a secondary or fixed experimental condition. Most DCF and NSAID nanocarriers reported to date are produced using conventional batch-based techniques, including thin-film hydration, solvent injection, and ionotropic gelation [16]. While these methods are widely accessible and experimentally convenient, they frequently suffer from batch-to-batch variability, limited control over particle size distribution, and poor scalability beyond laboratory scale. Consequently, both formulation composition and manufacturing methodology contribute to the key quality attributes used to evaluate formulation success, although the latter is rarely systematically analyzed as an independent variable. In recent years, this limitation has become increasingly evident with the emergence of advanced and more controllable manufacturing technologies. Continuous processing approaches, including microfluidic mixing, nanoprecipitation, supercritical fluid processing, electrospraying, and advanced drying techniques, have been progressively integrated with QbD principles and design-of-experiments (DoE) methodologies to improve process understanding, reproducibility, and scalability [17,18,19,20]. Among these, microfluidic-based systems are particularly notable for enabling precise control over particle size and PDI, improving batch consistency, and enhancing encapsulation efficiency, while also offering a more realistic pathway toward industrial-scale manufacturing [21,22,23,24]. This aspect is critical, as insufficient reproducibility and scalability remain major barriers in nanomedicine translation [25]. Despite extensive preclinical research activity, only a limited number of nanocarrier systems progress to clinical application, with manufacturing-related limitations representing a key contributor to this translational gap [26,27,28].

Although this review addresses nanocarrier fabrication strategies for NSAIDs as a therapeutic class, DCF is used as the principal reference compound throughout the analysis because it is among the most extensively studied NSAIDs in nanomedicine and provides the largest body of comparable formulation and biological data. For fabrication approaches where DCF-specific evidence remains limited, representative studies involving closely related NSAIDs are included to illustrate general manufacturing trends while explicitly indicating when surrogate data are used.

From a conceptual perspective, the performance of nanocarrier systems is determined by two complementary and interdependent components: carrier composition and fabrication strategy. Carrier-level effects arise from the intrinsic properties of the selected materials, including lipids, polymers, surfactants, and drug–excipient interactions, which collectively determine drug loading capacity, release behavior, biocompatibility, and biological functionality. In contrast, process-level effects are governed by the fabrication method and manufacturing conditions, influencing particle size distribution, polydispersity, encapsulation efficiency, batch-to-batch reproducibility, scalability, and process robustness. Although these two dimensions are closely interconnected, the influence of fabrication strategy has historically received less attention than carrier composition, despite its critical role in determining the quality attributes and translational potential of nanomedicine products.

Accordingly, this review focuses on fabrication strategy as an underrecognized but critical determinant of nanocarrier performance, while acknowledging its interdependence with formulation composition. Against this background, there remains a need for a structured analysis of DCF and NSAID nanocarrier systems that considers fabrication methodology alongside formulation composition and evaluates their combined influence on performance and manufacturability. The present review addresses this gap by emphasizing fabrication methodology as an important design variable that complements formulation composition in determining nanocarrier performance. Conventional batch methods, including chitosan-coated lipid vesicular systems, are considered as reference approaches, while emerging manufacturing technologies are critically evaluated in terms of encapsulation efficiency, particle size and polydispersity control, zeta potential, release behavior, reproducibility, and scalability. Through this comparative framework, the review aims to identify how advances in fabrication methodology, together with formulation design, contribute to meaningful improvements in DCF and NSAID nanomedicines and to clarify how current reporting practices may obscure parameters that are essential for clinical translation.

## 2. Scope and Method of the Review

This review aims to critically examine the role of fabrication methods as a key determinant of performance and translational potential in DCF and NSAID nanocarrier systems. Rather than focusing solely on carrier composition, the analysis reframes nanomedicine development through the lens of manufacturing strategy, highlighting how batch and emerging continuous processes influence critical quality attributes, including particle size, polydispersity, encapsulation efficiency, release behavior, reproducibility, and scalability. The scope is intentionally centered on nanoscale and submicron drug delivery systems reported in the literature between 2015 and 2026, with particular emphasis on recent advances in microfluidics, nanostructured lipid carriers, and QbD-guided formulation approaches. By integrating these developments within a unified comparative framework, the review seeks to identify which fabrication strategies offer genuine translational advantages and to clarify the extent to which manufacturing processes, rather than material selection alone, govern the clinical viability of NSAID nanomedicines.

### 2.1. Review Design and Reporting

This work was designed as a structured critical review rather than a systematic review or meta-analysis. Its primary objective is to organize and interpret a methodologically heterogeneous body of literature around a single analytical question: how the fabrication route governs the performance and translational potential of DCF and NSAID nanocarrier systems. Accordingly, the aim is not to generate pooled quantitative estimates, but to provide a comparative, method-oriented synthesis that highlights cross-platform trends in formulation performance and manufacturability.

To reduce subjectivity inherent to narrative synthesis, the review was conducted in accordance with the SANRA criteria [29]. These guidelines informed the overall structure of the review and ensured transparency in reporting, including an explicitly defined search strategy, predefined eligibility criteria, and the application of a fixed evaluation framework to all included studies. This framework enabled consistent appraisal of nanocarrier systems across key quality and translational attributes, as summarized in Table 2. Together, these methodological safeguards were implemented to enhance reproducibility, clarity, and analytical rigor while preserving the flexibility required for integrating a diverse and rapidly evolving literature base.

### 2.2. Information Sources and Search Strategy

PubMed/MEDLINE, Scopus, and the Web of Science Core Collection were searched for relevant studies published between January 2015 and February 2026, with greater emphasis placed on the 2022–2026 period to capture recent advances in nanocarrier fabrication while retaining earlier foundational literature for contextual relevance. The search was last updated in February 2026.

A structured search strategy was employed, combining terms related to the drug, therapeutic class, nanocarrier systems, and fabrication methodologies using database-specific Boolean operators. A representative search string included: (“diclofenac” OR “nonsteroidal anti-inflammatory” OR “NSAID” OR “ibuprofen” OR “naproxen” OR “ketoprofen” OR “indomethacin” OR “piroxicam”) AND (“nanoparticle*” OR “nanocarrier*” OR “liposome*” OR “nanostructured lipid carrier*” OR “solid lipid nanoparticle*” OR “polymeric nanoparticle*” OR “nanoemulsion*”) AND (“encapsulation” OR “fabrication” OR “preparation method” OR “microfluidic*” OR “nanoprecipitation” OR “thin-film hydration” OR “supercritical” OR “electrospray*” OR “spray-drying”) (PubMed: plain-text search; Scopus: TITLE-ABS-KEY(); Web of Science: TS = ()).

Eligibility was limited to peer-reviewed original research articles published in English. To ensure comprehensiveness, the reference lists of included studies and relevant review articles were manually screened to identify additional publications not captured in the primary database search.

### 2.3. Eligibility Criteria

The combined database search (PubMed/MEDLINE, Scopus, Web of Science Core Collection) returned 1787 records; after de-duplication by DOI and, where absent, by normalized title across all three databases, 1326 unique records were screened by title and abstract. Of these, 653 met the eligibility criteria below at the title/abstract level and were forwarded for full-text assessment, comprising 133 DCF-specific records and 520 records involving other NSAIDs. Full-text-level review confirmed 75 DCF-specific records as eligible primary data sources; the remainder were excluded, most commonly because DCF was mentioned only as a comparator rather than the loaded compound, or because the study described an analytical/detection method rather than a delivery system, or because no submicron/nanoscale carrier or quantitative formulation parameter was reported. Two records initially identified as DCF-specific were reclassified as surrogate-NSAID studies (aceclofenac, nabumetone) following full-text review. Given the scope of a structured narrative rather than a systematic review, the 522 surrogate-NSAID records were screened with proportionately lighter, though still criteria-based, scrutiny, yielding an estimated 300–320 eligible surrogate-NSAID studies. In total, this review is based on 75 DCF-specific studies and approximately 300–320 surrogate-NSAID studies, for an estimated total of 375–395 included studies.

Studies were considered eligible if they reported submicron- or nanoscale carrier systems loaded with DCF or other NSAIDs and included at least one quantitative formulation parameter, such as particle size, PDI, zeta potential, encapsulation efficiency, drug loading, or release behavior, together with a clearly identifiable fabrication method.

Studies were excluded if nanomaterials were used for analytical sensing, electrochemical detection, or environmental remediation purposes (e.g., adsorption or removal of DCF as an aqueous contaminant), as these applications fall outside the scope of drug delivery and encapsulation and represent a substantial but conceptually distinct body of literature. Review articles, opinion papers, and editorials were not considered as primary data sources but were used for contextual interpretation and validation of findings.

### 2.4. Evaluation Framework

To facilitate comparison between fabrication methods, seven manufacturing-related attributes were considered: particle size control, encapsulation performance, reproducibility, scalability, throughput, process complexity, and translational suitability. These attributes were assessed qualitatively based on evidence reported in the selected studies together with generally accepted engineering and pharmaceutical manufacturing principles. No numerical scoring system or weighting factors were applied, as the objective was to provide a structured comparative framework rather than a quantitative ranking of fabrication methods. The relative importance of each attribute should be interpreted in the context of the specific nanocarrier platform and intended pharmaceutical application.

Qualitative descriptors (high, moderate, or low) represent comparative assessments derived from the collective evidence available in the reviewed literature and should not be interpreted as standardized quantitative performance metrics.

To enable meaningful comparison across heterogeneous systems, each fabrication method was assessed using a consistent set of attributes capturing both product quality and manufacturability: (i) encapsulation efficiency and drug loading; (ii) particle size and its controllability; (iii) PDI as an indicator of size distribution homogeneity; (iv) zeta potential as a proxy for colloidal stability; (v) in vitro release behavior and associated kinetic modeling; (vi) batch-to-batch reproducibility; and (vii) scalability and process throughput, including the level of maturity toward continuous manufacturing and GMP compatible implementation [9,29,30,31].

The first five parameters correspond to the most frequently reported quality attributes in the primary literature, whereas reproducibility and scalability remain less consistently addressed despite their critical importance for translational performance. These latter parameters are particularly relevant for bridging the gap between laboratory-scale formulation development and clinical or industrial implementation.

Collectively, these seven attributes constitute the comparative framework used throughout this review. Within this framework, conventional batch-based methods, exemplified by chitosan-coated lipid vesicular systems, serve as the reference baseline against which emerging fabrication strategies are systematically evaluated.

## 3. Comparative Overview of Fabrication Methods

The strength of evidence supporting individual fabrication methods varies considerably across the reviewed literature. Conventional techniques, such as thin-film hydration, are supported by a larger body of experimental studies, whereas several emerging fabrication approaches are represented by a more limited number of reports, often using different nanocarrier compositions and experimental designs. Consequently, comparisons of reproducibility, scalability, and translational potential should be interpreted qualitatively and within the context of the available evidence rather than as definitive rankings.

**Figure 1:**
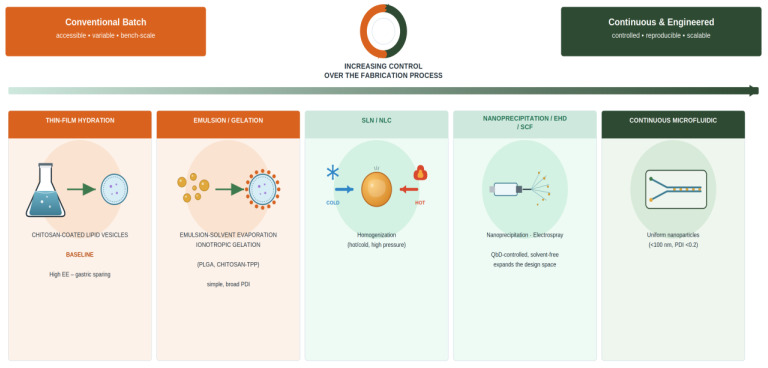
Fabrication of DCF and NSAID nanocarriers conceptualized as a continuum of process control. Conventional batch-based techniques (**left**), exemplified by chitosan-coated phosphatidylcholine vesicles used as the review’s baseline reference system (references [15,16]), transition toward continuous and engineered manufacturing approaches (**right**). Across the seven appraisal attributes, improvements in particle size control, PDI, batch-to-batch reproducibility, and scalability are observed along this continuum, whereas ease of implementation and the extent of existing in vivo validation remain key strengths of conventional batch methodologies. Quality by Design (QbD) provides the overarching methodological framework applied across all fabrication strategies. NLC, nanostructured lipid carrier; PDI, polydispersity index; EE, encapsulation efficiency.

The fabrication methods reported for DCF and other NSAID nanocarriers span a continuum from established bench-scale batch techniques to continuous, engineering-controlled processes (Figure 1).

Table 3 presents the comparative performance of DCF and related NSAID nanocarrier systems within the seven-attribute framework defined in Section 2.4, using thin-film hydration of chitosan-coated lipid vesicles as the reference system. The reported values are indicative ranges synthesized from the primary literature and are intended for cross-method comparison rather than absolute specification.

Table 3 summarizes the comparative performance of DCF and related NSAID nanocarrier systems across the seven-attribute framework, highlighting how fabrication method systematically influences both product quality and translational feasibility. Conventional batch methods, exemplified by thin-film hydration of chitosan-coated phosphatidylcholine vesicles, consistently deliver high encapsulation efficiency and biologically validated sustained release, including demonstrated in vivo anti-inflammatory efficacy, but remain constrained by operator dependence, broad size distributions, and limited scalability [15,16]. Similarly, PLGA-based nanoparticle systems prepared via emulsion diffusion or double emulsion techniques achieve nanoscale dimensions (≈90–150 nm) and sustained release profiles, yet reproducibility and manufacturing scalability remain moderate at best, even when improved through design-of-experiments optimization [11,13,14,39]. These findings reinforce that batch-derived systems can achieve strong pharmacological performance but often at the expense of process robustness.

In contrast, more recent and engineered manufacturing approaches demonstrate a progressive improvement in process control and scalability. Nanostructured lipid carriers (NLCs), particularly when optimized via Taguchi or similar statistical designs, combine sub-200 nm size control, high encapsulation efficiency, and improved reproducibility, while benefiting from established high-pressure homogenization infrastructure that supports industrial scalability [32,40]. Continuous microfluidic mixing further advances this trend by enabling precise control of nucleation under laminar flow conditions, yielding nanoparticles below 100 nm with narrow polydispersity (PDI < 0.2), low batch-to-batch variability, and seamless transition from screening to scale-up production [21,22,33,41]. These systems, therefore represent a transition from empirically controlled batch fabrication to continuously governed, design-space-defined manufacturing.

Additional emerging or hybrid approaches expand the formulation landscape but occupy intermediate positions within the translational spectrum. Electrospun fiber systems designed under full factorial QbD frameworks offer robust control over drug release behavior, though scalability remains moderate due to process constraints [34,42]. Supercritical fluid processing provides a solvent-free route with good scalability but typically yields micronized rather than true nanoscale particles for DCF [35]. Nanocrystal and nanosuspension technologies improve dissolution performance but remain limited by surfactant-dependent stability [36,43]. Likewise, ionotropic gelation and nanoemulsion-based systems achieve efficient drug loading and favorable physicochemical profiles, but their reproducibility and scale-up potential remain formulation- and process-dependent [37,38,44]. Collectively, these data position fabrication method as a primary determinant of nanocarrier performance rather than a secondary procedural detail.

## 4. Conventional Batch Fabrication as the Baseline Reference

Any rigorous appraisal of the current direction of the field must begin with its prevailing state. The DCF and NSAID nanocarrier literature is, to a large extent, a literature of batch-based fabrication: thin-film hydration of phospholipid vesicles, ethanol or solvent injection, emulsion solvent evaporation, and ionotropic gelation collectively account for the vast majority of systems reported over the past three decades. These methods are not historical artifacts; rather, they remain widely used due to their accessibility, low cost, and tolerance to modest infrastructure. Importantly, they consistently reproduce the key pharmaceutical objectives that motivate nanoencapsulation, namely improved apparent solubility of poorly water-soluble Biopharmaceutics Classification System (BCS) class II drugs, prolonged duration of action, and a reduction in gastrointestinal toxicity associated with free DCF [1,9].

Our previously published work is representative of this established baseline and is presented here as an exemplar rather than a novel contribution [15,16]. Using a thin-film hydration approach, we developed phosphatidylcholine vesicles loaded with DCF sodium and stabilized via a chitosan coating. This coating improved colloidal stability, imparted a positive surface charge, and enabled long-term physical stability of the dispersion for up to six months at 4 °C. The system achieved high encapsulation efficiency and sustained in vitro release compared with free DCF, while demonstrating hemocompatibility in vitro and biocompatibility in vivo, without relevant hematological, biochemical, or histopathological alterations in liver or kidney tissues. Most importantly for an NSAID formulation, the chitosan-stabilized vesicles significantly reduced DCF-induced gastric mucosal damage. Pharmacodynamically, the formulation produced a delayed onset but sustained antinociceptive effect in tail-flick and hot-plate models, alongside prolonged anti-inflammatory activity in carrageenan-induced paw edema and cotton-pellet granuloma models, thereby confirming that the benefit of sustained release was translated in vivo rather than inferred solely from in vitro dissolution behavior [15,16].

This point is critical, as it highlights one of the enduring strengths of the batch-based literature: the depth and robustness of biological validation. Indeed, many comparable systems, including PLGA-based DCF carriers and related polymeric platforms, have been evaluated across hemocompatibility, multi-organ biochemical safety, histopathology, and multiple in vivo efficacy models [11,12,13,45]. However, the principal limitation of these approaches is structural rather than mechanistic. Batch techniques such as thin-film hydration are inherently sensitive to operator and process conditions because critical steps, including lipid film formation, hydration, and size reduction, are frequently performed manually. Although the implementation of standardized operating procedures, tighter control of critical process parameters, and QbD principles can substantially reduce variability, complete elimination of operator dependence remains difficult due to the intrinsic characteristics of batch processing. Consequently, particle size distribution, polydispersity, and batch-to-batch reproducibility may still be more variable than in continuous manufacturing platforms [9]. Moreover, post-processing steps required for storage stability, such as vesicle drying and reconstitution, complicate translation to scalable oral or parenteral products. Most critically, scalability remains limited. The very attributes used to define formulation success, particle size, PDI, zeta potential, and encapsulation efficiency, are themselves strongly dependent on the fabrication route, yet this route is rarely treated as an independent experimental variable. It is precisely this limitation that emerging manufacturing strategies in recent years have begun to address.

## 5. Emerging Fabrication Strategies (2022–2026)

Conventional methods are characterized by high accessibility at the expense of precise control, whereas the approaches that have gained prominence over the review period are defined by the opposite trade-off: they introduce a higher degree of engineering control into the fabrication process, thereby transforming key quality attributes, previously emergent and variable, into parameters that can be systematically designed, tuned, and reproduced. Four principal developments can be identified, which are best understood not as competing strategies but as points along a continuum of progressively increasing process control.

### 5.1. Microfluidic Continuous Manufacturing of Nanocarriers

Microfluidic mixing represents the most consequential shift among emerging fabrication strategies, as it directly addresses the intrinsic limitations of batch processing at its origin. By bringing an organic lipid or polymer phase into contact with an aqueous antisolvent under laminar, geometrically defined flow conditions, microfluidic systems decouple nanoparticle formation from operator-dependent variables and instead render nucleation a function of precisely tunable parameters, primarily total flow rate and flow-rate ratio, rather than empirical agitation or hydration conditions [17,21].

This shift translates into substantially improved control over particle size and polydispersity. Vesicular systems and lipid nanoparticles below 100 nm with PDI below 0.2 are now routinely achievable, and the integration of DoE and machine learning–based optimization has further transformed parameter selection from empirical adjustment into a predictive design space [22,33,41]. However, the most important advantage of microfluidic manufacturing is not merely improved nanoscale uniformity, but process continuity. Parallelized and structured micromixer platforms enable high-throughput formulation screening at sub-milliliter scale while maintaining seamless transition to continuous production, with consistently low batch-to-batch coefficients of variation. This combination of reproducibility and scalability directly addresses two of the most critical yet often unmet criteria identified in the seven-attribute framework [19,23].

It should be noted that DCF-specific applications of microfluidic manufacturing remain relatively limited, with most advances driven by lipid nanoparticle platforms for nucleic acid delivery and oncology therapeutics. Rather than representing a limitation, this gap should be interpreted as an opportunity, as the well-established chitosan-coated lipid vesicle systems validated in batch processing are, in principle, directly transferable to continuous microfluidic platforms, thereby combining proven biological performance with enhanced manufacturing control.

Despite these advantages, several challenges continue to limit the widespread implementation of microfluidic manufacturing. The relatively low volumetric flow rates of many laboratory-scale devices may require parallelization for industrial production. In addition, the high degree of formulation dilution often necessitates downstream concentration steps, while solvent compatibility and channel fouling may restrict the range of formulations that can be processed. Furthermore, the specialized equipment and technical expertise required for microfluidic systems may limit accessibility, particularly in research environments with limited infrastructure.

### 5.2. Nanostructured and Solid–Liquid Lipid Carriers: Evolution of Lipid Matrix Design

A second advance, more incremental in conceptual novelty but already industrially mature, concerns redesign of the lipid matrix itself. Nanostructured lipid carriers (NLCs), in which a liquid lipid is incorporated into a solid lipid core to generate a deliberately imperfect crystalline lattice, were developed to overcome two well-recognized limitations of first-generation solid lipid nanoparticles (SLNs), namely restricted drug loading capacity and drug expulsion during storage. Comparative studies consistently report that NLCs provide improved encapsulation efficiency, enhanced colloidal stability, and reliable control over particle sizes below 200 nm [9,10].

For DCF, this approach extends beyond theoretical relevance. A recent study (2025) reported the formulation of DCF sodium-loaded NLCs, optimized via a Taguchi experimental design, followed by comprehensive physicochemical characterization and in vivo evaluation of anti-inflammatory activity. The system was explicitly designed to address the dissolution limitations associated with Biopharmaceutics Classification System (BCS) class II drugs [32,40].

Because NLCs are typically produced via melt-emulsification followed by high-pressure homogenization, processes already established at industrial scale in both pharmaceutical and cosmetic manufacturing, they represent a pragmatic intermediate category within Table 2. They combine a relatively high degree of reproducibility and scalability with the biological familiarity and regulatory tractability of lipid-based carrier systems.

Although NLCs overcome several limitations associated with conventional solid lipid nanoparticles, they also present formulation and manufacturing challenges. The incorporation of mixed solid and liquid lipid matrices increases formulation complexity and requires careful optimization to ensure long-term physical stability and batch-to-batch reproducibility. In addition, lipid loss during processing and storage, together with the selection of compatible lipid combinations, may influence encapsulation efficiency, drug release behavior, and overall product stability.

### 5.3. Nanoprecipitation, Electrohydrodynamic, and Supercritical Fluid Processing for Nanocarrier Fabrication

Around these two leading approaches sits a cluster of complementary techniques, each addressing a specific facet of the manufacturability challenge. Nanoprecipitation provides operational simplicity and a natural route towards semi-continuous processing. Electrohydrodynamic techniques, including electrospraying and electrospinning, have been applied to NSAIDs under explicit QbD frameworks: a full-factorial study of indomethacin- and DCF-loaded electrospun fibrous patches defined a quality target product profile, identified applied voltage and needle-to-collector distance as critical process parameters, and mapped a design space against measured fiber characteristics, demonstrating that even an unconventional route can be rendered tractable when approached as an engineered process rather than a craft [34,42]. Supercritical fluid processing offers a solvent-free and inherently GMP-aligned alternative that circumvents residual-solvent concerns associated with emulsion-based methods, although for DCF, reported rapid expansion processes have thus far yielded micronized rather than truly nanoscale particles [35].

Nanomilling and microfluidization remain pragmatic options where a DCF nanocrystal, rather than a carrier-based encapsulation system, is the intended product, with demonstrated improvements in dissolution rate for this BCS Class II compound [36]. None of these constitutes a universal solution; rather, their value lies in expanding the available design space within which a formulator can balance encapsulation performance against stability, residual solvent burden, and scalability constraints.

### 5.4. Quality by Design as the Unifying Framework for Nanocarrier Fabrication

These otherwise disparate fabrication methods are unified less by a shared technological basis than by a common epistemological framework. The QbD paradigm, linking a Quality Target Product Profile (QTPP) to Critical Quality Attributes (CQAs), and subsequently to Critical Material Attributes (CMAs) and Critical Process Parameters (CPPs) through formal DoE and ICH Q8-defined design space methodologies, has become the central organizing principle of modern nanocarrier development. Its widespread adoption is precisely what enables fabrication routes to be meaningfully discussed as determinants of performance rather than as purely procedural details [20,24].

For DCF and related NSAIDs, factorial and response surface methodologies have been extensively applied to optimize key CQAs, including particle size, surface charge, encapsulation efficiency, and release kinetics across chitosan-, PLGA-, and lipid-based systems. These approaches replace one-factor-at-a-time empiricism with multivariate statistical models capable of quantifying interactions between variables, thereby improving both reproducibility and scalability [20,37,46]. The most recent evolution of this paradigm is the integration of computational modeling and machine learning, often referred to as Quality-by-Digital-Design, which aims to reduce experimental burden while further enhancing process predictability and batch consistency [33].

From a translational perspective, this shift implies that statistical experimental design is no longer an optional optimization step but the primary mechanism by which claims of robustness and clinical readiness are substantiated. Importantly, this regulatory logic is now extending beyond experimental design into manufacturing modality itself. The introduction of ICH Q13 on continuous manufacturing of drug substances and drug products (effective across major regulatory regions since 2023) provides a formal regulatory framework for the continuous processes toward which microfluidic and related technologies naturally converge, complementing the risk-management principles outlined in ICH Q9 [47,48]. For DCF and NSAID nanocarriers, this alignment has concrete implications: inherently continuous fabrication strategies are not only more reproducible in principle but are increasingly congruent with the regulatory pathways required for scalable clinical translation.

Taken together, the emerging fabrication technologies discussed in this review should be viewed as direct responses to the principal limitations of conventional batch-based manufacturing rather than as entirely new formulation paradigms. Traditional techniques, particularly thin-film hydration, remain valuable because of their simplicity, flexibility, and extensive experimental validation; however, they are inherently associated with operator-dependent processing, limited control over mixing conditions, broad particle size distributions, and challenges related to batch-to-batch reproducibility and industrial scale-up. In contrast, continuous microfluidic manufacturing provides precise control over fluid dynamics and nanoparticle self-assembly, resulting in improved particle size uniformity, reduced process variability, and seamless scalability. Similarly, nanostructured lipid carriers address the limited drug-loading capacity and drug expulsion observed with conventional solid lipid nanoparticles through rational redesign of the lipid matrix. More broadly, the integration of QbD, DoE, and, more recently, machine learning shifts formulation development from empirical optimization toward predictive, data-driven process control. Collectively, these advances not only improve the physicochemical characteristics of nanocarriers but also address the manufacturing challenges that have historically limited their clinical translation, including reproducibility, scalability, process robustness, and regulatory readiness.

It is important to distinguish between technological innovation and industrial maturity when evaluating nanocarrier fabrication methods. High-pressure homogenization is an established manufacturing technology that has demonstrated industrial scalability, regulatory acceptance, and commercial applicability for lipid-based nanocarriers, including NLCs. In contrast, although continuous microfluidic manufacturing offers significant advantages in terms of process control, particle size uniformity, and reproducibility, many implementations remain at the laboratory or pilot scale. Consequently, further optimization, process standardization, and validation under industrial manufacturing conditions are required before widespread commercial adoption can be achieved.

## 6. The Fabrication Method as a Critical Determinant of Translational Performance

The argument of this review is ultimately a translational one, and the translational record remains sobering. From a translational perspective, the available evidence supporting nanocarrier fabrication methods should be interpreted within a hierarchical framework. Initial in vitro studies provide essential information regarding physicochemical characteristics, drug release behavior, and cytocompatibility, whereas ex vivo models offer additional insight into tissue permeation and local drug delivery. In vivo investigations further establish pharmacokinetic performance, therapeutic efficacy, and safety under physiological conditions. However, successful clinical translation requires an additional level of validation beyond biological performance, namely manufacturing readiness. This encompasses reproducibility, process robustness, scalability, quality control, and compatibility with regulatory and Good Manufacturing Practice (GMP) requirements. Consequently, fabrication methods should be evaluated not only according to their biological outcomes but also according to their capacity to support reliable and industrially feasible production.

Despite a preclinical literature comprising thousands of nanomedicine candidates, one recent estimate suggests that only approximately 50–80 nanomedicines had achieved regulatory approval for clinical use worldwide by 2025, highlighting the persistent gap between preclinical development and clinical translation [25]. The few enduring clinical successes, such as liposomal doxorubicin and albumin-bound paclitaxel, are repeatedly cited precisely because they remain exceptions rather than the rule [25,27]. The principal barriers separating promising formulations from clinically viable medicines are not generally related to insufficient pharmacological activity, but rather arise from the bio–nano interface, immunogenicity, and, most critically, limitations in scalability and batch-to-batch manufacturing reproducibility [25,27].

This disconnect is amplified by prevailing reporting conventions in the field. The five physicochemical descriptors that dominate the primary literature, encapsulation efficiency, particle size, PDI, zeta potential, and release behavior, are essential but inherently batch-specific, reflecting properties of individual laboratory-scale preparations. In contrast, the two parameters that ultimately govern translational success, process reproducibility and scalability, are reported far less consistently. As a result, a formulation may appear optimal based on conventional quality metrics while remaining fundamentally non-translatable. Reframing the fabrication method as a central analytical lens makes this limitation explicit, since reproducibility and scalability are not intrinsic properties of the carrier system itself, but emergent properties of the manufacturing process [25].

In the context of DCF and NSAIDs, these translational constraints are particularly pronounced. Unlike oncology nanomedicines, these are low-cost, high-volume therapeutics used in predominantly benign or chronic conditions, where the rationale for nanoformulation lies in incremental improvements in safety and tolerability rather than transformative gains in survival. Under these conditions, a nanocarrier that cannot be manufactured reproducibly, at scale, and at acceptable cost is not merely difficult to translate but effectively fails its core value proposition. Within this framework, the continuous and QbD-driven manufacturing approaches discussed above should not be viewed as incremental refinements, but as strategies that directly address the very criteria upon which NSAID nanomedicines will ultimately be judged.

Two downstream processing steps further illustrate this translational gap, as they are rarely emphasized in formulation studies yet are routinely decisive for clinical applicability: sterilization and lyophilization. Most lipid- and polymer-based nanocarriers are incompatible with terminal sterilization methods such as autoclaving or irradiation, which often compromise particle size distribution, structural integrity, and drug payload. As a result, sterile filtration or aseptic processing is required, approaches that may be manageable at laboratory scale but become increasingly challenging under industrial manufacturing constraints.

Similarly, lyophilization, which is frequently required to ensure long-term stability of nanosuspensions, introduces additional sources of variability. Freeze–thaw stresses and cryoprotectant selection can significantly alter particle size, polydispersity, and reconstitution behavior, making tight process control essential for maintaining critical quality attributes. Importantly, both sterilization and lyophilization are substantially more robust when integrated into continuous, design-controlled manufacturing workflows than when appended as post hoc steps to inherently variable batch processes. This further reinforces the central premise of this review: that translational success is governed less by carrier design alone and more by the method through which the system is manufactured.

## 7. Limitations

Despite its structured and method-driven approach, several limitations should be acknowledged when interpreting the conclusions of this review. First, the study design is narrative rather than systematic; although it follows SANRA guidelines and applies predefined eligibility criteria and a consistent appraisal framework, it does not include exhaustive protocol-driven screening or formal risk-of-bias assessment. As a result, some degree of selection bias cannot be fully excluded, particularly given the emphasis on recent literature and the inclusion of representative rather than statistically comprehensive datasets.

Second, the comparative synthesis relies on heterogeneous primary studies that differ substantially in experimental design, reporting depth, and endpoint selection. Many of the included works were optimized for formulation development rather than cross-platform comparison, leading to variability in how key parameters such as particle size distribution, encapsulation efficiency, or release kinetics are measured and reported. In some cases, indirect comparisons were required, particularly when DCF-specific data were limited and closely related NSAID systems were used as surrogates.

Third, although the seven-attribute framework enables structured evaluation across fabrication methods, not all attributes are equally reported across the literature. In particular, scalability, batch-to-batch reproducibility, and manufacturing throughput are frequently underreported, which may limit the granularity of cross-method comparisons and introduce uncertainty into translational interpretations.

Fourth, the review focuses primarily on formulation and manufacturing aspects and does not systematically address in-depth pharmacokinetic, pharmacodynamic, or long-term toxicological differences across all nanocarrier classes. While selected in vivo findings are discussed where available, these data are not sufficiently uniform to support formal comparative efficacy conclusions.

Finally, the rapid evolution of nanomanufacturing technologies means that some emerging methods, particularly in continuous processing, microfluidics, and digitally guided formulation design, may be underrepresented due to publication lag. Consequently, the conclusions should be interpreted as reflective of the current state of the field within the defined search window rather than as a definitive or exhaustive mapping of all existing technologies.

Another limitation of the present review is the heterogeneous nature of the available evidence regarding manufacturing performance. Parameters such as reproducibility, scalability, and production throughput are inconsistently reported across studies and are frequently assessed using different experimental conditions and evaluation criteria. This heterogeneity limits direct comparisons between fabrication methods and highlights the need for standardized reporting of manufacturing-related outcomes in future nanocarrier research.

Overall, these limitations do not undermine the central premise of the review but rather contextualize its interpretative scope and reinforce the need for more standardized, manufacturing-oriented reporting in future nanocarrier studies.

## 8. Conclusions

Read collectively, the literature on DCF and other NSAID nanocarriers demonstrates that nanocarrier performance is governed by the interplay between formulation composition and fabrication strategy. While carrier composition defines the intrinsic physicochemical and biological characteristics of the system, the fabrication process determines how consistently these attributes can be achieved, reproduced, and translated into clinically relevant products. Consequently, fabrication should be regarded as a critical design variable rather than merely a downstream manufacturing step.

Conventional fabrication methods, particularly thin-film hydration and high-pressure homogenization, continue to play a fundamental role in nanocarrier development owing to their established performance, extensive experimental validation, and, in some cases, industrial maturity. However, their inherent limitations, including operator dependence, batch-to-batch variability, and restricted process control, may constrain large-scale manufacturing and clinical translation. In contrast, emerging fabrication technologies, including continuous microfluidics, electrohydrodynamic processing, supercritical-fluid techniques, and advanced lipid engineering approaches such as nanostructured lipid carriers, offer promising opportunities to improve process control, reproducibility, and manufacturing robustness. Nevertheless, the level of experimental and industrial validation currently differs considerably among these technologies, and further comparative studies are required before broad generalization across all NSAID nanocarrier platforms can be made.

The evidence synthesized in this review further suggests that successful translation of NSAID nanomedicines depends not only on demonstrating favorable physicochemical properties and biological performance but also on ensuring reproducible, scalable, and regulatory-compliant manufacturing processes. From a practical perspective, no single fabrication method can be considered universally optimal. Instead, the selection of an appropriate manufacturing platform should be guided by the intended therapeutic application, the characteristics of the active pharmaceutical ingredient, the desired release profile, and the anticipated requirements for scalability, quality assurance, cost-effectiveness, and regulatory implementation.

This review supports the view that advances in NSAID nanomedicine will be driven not only by the development of innovative carrier systems but also by the integration of robust manufacturing strategies capable of bridging the persistent gap between laboratory-scale formulation research and clinically viable pharmaceutical products. Future progress will therefore rely on combining rational formulation design with advanced, reproducible, and industrially feasible fabrication technologies to accelerate the successful clinical translation of nanocarrier-based NSAID therapies.

Importantly, technological innovation should not be equated with industrial readiness, as fabrication methods differ considerably in their level of manufacturing maturity and regulatory validation.

## 9. Perspectives

From the authors’ perspective, future progress in NSAID nanomedicine will depend less on the discovery of entirely new carrier materials than on the ability to manufacture existing systems reproducibly, economically, and at a clinically relevant scale. While conventional batch techniques remain indispensable during early-stage formulation development because of their simplicity and flexibility, they are unlikely to satisfy the manufacturing robustness required for widespread clinical implementation. Emerging fabrication technologies should therefore be viewed not merely as alternative production methods but as enabling platforms that integrate precise process control, QbD principles, and continuous manufacturing into the development workflow. Consequently, selection of a fabrication strategy should be based not only on the desired physicochemical characteristics of the nanocarrier but also on its anticipated translational potential, including reproducibility, scalability, regulatory compatibility, and cost-effective manufacturing.

The future development of NSAID nanomedicines is likely to depend less on the discovery of entirely new carrier materials than on the ability to manufacture existing nanocarrier systems with the reproducibility, scalability, and process robustness required for successful clinical translation. While conventional batch-based fabrication methods remain indispensable for proof-of-concept studies because of their simplicity, versatility, and extensive experimental validation, their inherent limitations, including operator dependence, batch-to-batch variability, and restricted scalability, highlight the need for manufacturing strategies that are better aligned with industrial production and regulatory expectations.

To accelerate translation, several research priorities should be addressed. First, standardized metrics and reporting criteria should be established for evaluating manufacturing reproducibility, process robustness, and batch-to-batch consistency, enabling meaningful comparisons across fabrication platforms. Second, direct side-by-side comparisons between conventional batch processes and emerging continuous manufacturing approaches should be performed using identical carrier compositions and standardized experimental conditions to isolate the specific contribution of the fabrication process to nanoparticle quality and biological performance. Third, future studies should routinely report manufacturing-related parameters, including process yield, production throughput, downstream processing requirements, scalability, long-term stability, sterilization compatibility, and lyophilization performance, in addition to conventional physicochemical characterization. Fourth, broader implementation of QbD principles, supported by process analytical technologies and digital modeling, should facilitate more rational process optimization and improve manufacturing consistency. Finally, emerging technologies such as continuous microfluidic manufacturing should be validated under industrially relevant conditions to establish their technical, economic, and regulatory feasibility for large-scale pharmaceutical production.

From the authors’ perspective, fabrication strategy should no longer be regarded as a downstream technical consideration but as a fundamental design variable that acts in concert with formulation composition to determine nanocarrier quality, manufacturability, and translational success. Consequently, selecting an appropriate fabrication platform should involve balancing formulation performance with reproducibility, scalability, cost-effectiveness, and regulatory compliance. Future advances in NSAID nanomedicine will therefore rely not only on innovative carrier design but also on the integration of robust, standardized, and industrially feasible manufacturing strategies capable of bridging the persistent gap between promising laboratory formulations and clinically viable therapeutic products.

## Figures and Tables

**Table 1:** Representative NSAIDs, conventional formulations, major limitations, and the rationale for nanocarrier-based delivery systems.

NSAID	Common Marketed Formulations	Major Limitations of Conventional Formulations	Potential Advantages of Nanocarrier Systems
Diclofenac	Tablets, capsules, injections, topical gel	Poor aqueous solubility, short half-life, gastrointestinal, renal and cardiovascular toxicity	Improved solubility, sustained release, reduced systemic exposure, improved safety
Ibuprofen	Tablets, capsules, suspensions	Poor solubility, frequent dosing, gastrointestinal irritation	Enhanced dissolution, prolonged release, lower dosing frequency
Naproxen	Tablets, suspensions	Delayed dissolution, gastrointestinal toxicity, prolonged systemic exposure	Controlled release, improved bioavailability, reduced GI irritation
Ketoprofen	Tablets, topical gel	Poor water solubility, photosensitivity, gastrointestinal adverse effects	Improved solubility, targeted delivery, reduced systemic toxicity
Indomethacin	Capsules, suppositories	Severe gastrointestinal and CNS adverse effects, poor tolerability	Sustained release, reduced peak plasma concentrations, improved tolerability
Piroxicam	Capsules, tablets	Long half-life, gastrointestinal ulceration, poor aqueous solubility	Controlled drug release, improved therapeutic index, reduced adverse effects

**Table 2:** Self-assessment of the present review against the SANRA. Each of the six items is scored from 0 to 2 (maximum total 12).

SANRA Item	Score (0–2)	Justification
1. Justification of the article’s importance for the readership	2	The review addresses a clearly defined gap in the current literature, namely that most DCF and NSAID nanocarrier studies focus primarily on optimizing carrier composition, while treating the fabrication method as a fixed experimental background. In contrast, the present work reframes the encapsulation route as a key determinant of nanocarrier performance and translational potential, a perspective of direct relevance to both formulation scientists and clinical researchers.
2. Statement of concrete aims or formulation of questions	2	A specific objective is articulated in the Introduction: to position conventional batch-based techniques as the reference baseline, to critically evaluate emerging manufacturing approaches, and to compare them using a predefined seven-attribute framework in order to identify which methodological advancements translate into improved DCF and NSAID nanomedicines.
3. Description of the literature search	1	The literature search strategy is transparently reported, including the databases consulted (PubMed/MEDLINE, Scopus, Web of Science), the time frame (2015–2026, with emphasis on 2022–2026), a representative Boolean search string, language restrictions, and eligibility criteria. In line with the narrative and non-systematic design, the search was intentionally recency-weighted and supplemented by manual screening of reference lists rather than following a fully exhaustive systematic review protocol.
4. Referencing	2	All key statements are supported by appropriate primary and secondary sources, each accompanied by DOI identifiers where available. Where evidence is extrapolated from closely related NSAID systems rather than DCF-specific data, this is explicitly acknowledged.
5. Scientific reasoning (e.g., incorporation of appropriate evidence)	2	The analytical framework is applied consistently across all included fabrication methods, enabling structured comparison. Quantitative performance data are synthesized from the selected studies, while translational relevance is discussed in relation to reported scale-up and approval trends rather than speculative inference.
6. Appropriate presentation of data and findings	2	Comparative results are presented in a structured table with per-entry citations and clearly stated limitations, including indicative ranges and NSAID surrogates where DCF-specific data are limited. A schematic representation further summarizes the methodological continuum. Importantly, no unsupported claims are made regarding DCF-specific performance in microfluidic systems, ensuring methodological and factual rigor throughout.
Total	11/12	A SANRA score of 11/12 indicates a high-quality narrative review. The single criterion not fully met reflects the intentionally recency-weighted and non-systematic search strategy, which was adopted in accordance with the specific scope and design of this review.

SANRA criteria were applied as defined by Baethge et al. [29]. The assessment was conducted by the authors and is reported to support the transparency and methodological rigor of this narrative review.

**Table 3:** Comparative overview of representative fabrication methods and selected case studies included in this structured narrative review. Numerical physicochemical parameters are reported directly from the original studies whenever available. Qualitative descriptors represent comparative assessments based on the collective evidence reported in the literature and are intended to facilitate methodological comparison rather than provide a formal ranking of fabrication methods. Owing to differences in nanocarrier composition, experimental endpoints, and study design, comparisons between methods should be interpreted as indirect unless otherwise stated. Where DCF-specific evidence was unavailable, representative studies involving other NSAIDs were included and are explicitly identified.

Method (Drug)	EE/Loading	Size and PDI	Zeta/Stability	In Vitro Release	Reproducibility	Scalability/ Throughput
Thin-film hydration; chitosan-coated PC vesicles (DCF sodium) [15,16]	H modified release	Microvesicle scale; broad PDI	Chitosan-positive; stable ≥ 6 months (4 °C)	Prolonged; in vivo anti-inflammatory confirmed	Operator-dependent	Limited; Batch
PLGA NPs, emulsion-diffusion (DCF) [11]	EE 77–80%	92–108 nm	−11 to −28 mV	Sustained	M	M
PLGA NPs, chitosan-coated (DCF sodium) [13]	H	399–404 nm	−14 to +27 mV	pH-responsive	M	M
PLGA NPs, double-emulsion + DoE (DCF sodium) [14]	H	~150 nm; monodisperse	Negative	Sustained	M Good (DoE)	M
NLC-in-gel, Taguchi (DCF sodium) [32]	H	Sub-200 nm	Negative	Prolonged; BCS-II dissolution improved	H Good (Taguchi)	H HPH mature
Continuous microfluidic mixing (lipid NPs/liposomes) [21,22,33]	H	<100 nm; PDI < 0.2	Tightly controlled	Tunable; low burst	H low batch coefficient of variation	H screening to liter-scale
† Electrospun fibers, full-factorial QbD (indomethacin + DCF sodium) [34]	H (solid matrix)	Fiber < 1000 nm	n/a (solid matrix)	Voltage/distance-tunable	M Good under DoE	M
Supercritical RESS, scCO_2_ (DCF) [35]	n/a (drug particles)	1.3–10.9 µm	—	Dissolution-enhanced	H	H solvent-free
Nanocrystal/nanosuspension (DCF) [36]	n/a (crystalline)	Nanocrystal	Surfactant-dependent	Dissolution ≈65% vs. ~50%	H	H
† Ionotropic gelation, chitosan–TPP (indomethacin) [37]	Efficient (Box–Behnken)	321–675 nm	+25 to +32 mV	Sustained	M	L
Nanoemulsion/nanoemulgel (DCF sodium) [38]	H (solubilized)	64–200 nm; PDI ~0.24	−30 to −39 mV	Enhanced permeation	M	M (low-energy)
Thin-film hydration; chitosan-coated PC vesicles (DCF sodium) [15,16]	H modified release	Microvesicle scale; broad PDI	Chitosan-positive; stable ≥ 6 months (4 °C)	Prolonged; in vivo anti-inflammatory confirmed	Operator-dependent	Limited; Batch
PLGA NPs, emulsion-diffusion (DCF) [11]	EE 77–80%	92–108 nm	−11 to −28 mV	Sustained	M	M
PLGA NPs, chitosan-coated (DCF sodium) [13]	H	399–404 nm	−14 to +27 mV	pH-responsive	M	M
PLGA NPs, double-emulsion + DoE (DCF sodium) [14]	H	~150 nm; monodisperse	Negative	Sustained	M Good (DoE)	M
NLC-in-gel, Taguchi (DCF sodium) [32]	H	Sub-200 nm	Negative	Prolonged; BCS-II dissolution improved	H Good (Taguchi)	H HPH mature
Continuous microfluidic mixing (lipid NPs/liposomes) [21,22,33]	H	<100 nm; PDI < 0.2	Tightly controlled	Tunable; low burst	H low batch CV	H screening to liter-scale
† Electrospun fibers, full-factorial QbD (indomethacin + DCF sodium) [34]	H (solid matrix)	Fiber < 1000 nm	n/a (solid matrix)	Voltage/distance-tunable	M Good under DoE	M
Supercritical RESS, scCO_2_ (DCF) [35]	n/a (drug particles)	1.3–10.9 µm	—	Dissolution-enhanced	H	H solvent-free
Nanocrystal/nanosuspension (DCF) [36]	n/a (crystalline)	Nanocrystal	Surfactant-dependent	Dissolution ≈65% vs. ~50%	H	H
† Ionotropic gelation, chitosan–TPP (indomethacin) [37]	Efficient (Box–Behnken)	321–675 nm	+25 to +32 mV	Sustained	M	L
Nanoemulsion/nanoemulgel (DCF sodium) [38]	H (solubilized)	64–200 nm; PDI ~0.24	−30 to −39 mV	Enhanced permeation	M	M (low-energy)

Abbreviations: H = high; M = moderate; L = low. Qualitative ratings reflect comparative assessments derived from the reviewed literature and pharmaceutical manufacturing principles and should not be interpreted as standardized quantitative performance metrics. † Representative non-DCF NSAID study included because no DCF-specific evidence was identified for this fabrication method.

## Data Availability

No new datasets were generated during this study. The data supporting the findings of this review are derived from published articles cited in the reference list. Therefore, data sharing is not applicable.
